# Cirrhotic Endothelial Progenitor Cells Enhance Liver Angiogenesis and Fibrosis and Aggravate Portal Hypertension in Bile Duct-Ligated Cirrhotic Rats

**DOI:** 10.3389/fphys.2020.00617

**Published:** 2020-06-11

**Authors:** Dinesh Mani Tripathi, Mohsin Hassan, Hamda Siddiqui, Impreet Kaur, Preety Rawal, Chaggan Bihari, Savneet Kaur, Shiv K. Sarin

**Affiliations:** ^1^Department of Molecular and Cellular Medicine, Institute of Liver and Biliary Sciences, New Delhi, India; ^2^School of Biotechnology, Gautam Buddha University, Greater Noida, India; ^3^Department of Pathology, Institute of Liver and Biliary Sciences, New Delhi, India; ^4^Department of Hepatology, Institute of Liver and Biliary Sciences, New Delhi, India

**Keywords:** endothelial progenitor cells, fibrosis, angiogenesis, portal hypertension, cell transplant, vascular endothelial growth factor

## Abstract

**Background:**

Circulating cirrhotic endothelial progenitor cells (EPC) interact with both liver sinusoidal endothelial cells (LSEC) and hepatic stellate cells (HSC) and promote angiogenesis *in vitro*. This study evaluated the effect of cirrhotic and control EPCs on hepatic angiogenesis, microcirculation, and fibrosis *in vivo* in rat models of cirrhosis.

**Methodology:**

Animal models of cirrhosis were prepared by bile duct ligation (BDL). Circulating EPCs isolated from healthy human and cirrhotic blood were characterized by flow cytometry, cultured and administered through the tail vein in BDL rats after 2 weeks of ligation. The cells were given thrice a week for 2 weeks. The untreated group of BDL rats received only saline. Fibrosis was evaluated by Masson’s trichrome staining. Dedifferentiated LSECs were identified by the expression of CD31, and activated HSCs were marked as alpha-SMA-positive cells and were studied by immunohistochemistry and western blotting in saline-, healthy EPC-, and cirrhotic EPC-treated rats. *In vivo*, hepatic and systemic hemodynamic parameters were evaluated. Liver functions were evaluated.

**Results:**

In comparison to controls, BDL rats revealed an increase of fibrosis and angiogenesis. Among the treated rats, cirrhotic EPC-treated rats had increased fibrosis grade as compared to healthy EPC-treated and saline-treated rats. There was an increase of both fibrosis and angiogenesis markers, alpha-SMA and CD31 in cirrhotic EPC-treated rats as compared to healthy EPC-treated and saline-treated rats in immunohistochemistry and western blot studies. Cirrhotic EPC-treated BDL rats had high portal pressure and portal blood flow with significantly elevated hepatic vascular resistance in comparison with healthy EPC- and saline-treated BDL animals, without significant differences in mean arterial pressure. Cirrhotic EPC-treated BDL rats also showed a substantial increase in the hepatic expression of angiogenic receptors, VEGFR2 and CXCR4 in comparison with saline-treated rats.

**Conclusion:**

The study suggests that transplantation of cirrhotic EPCs enhances LSEC differentiation and angiogenesis, activates HSCs and worsens fibrosis, thus resulting in hepatic hemodynamic derangements in BDL-induced cirrhosis.

## Introduction

Angiogenesis is the process of formation of new blood vessels from pre-existing vasculature and mature endothelial cells. In liver, physiological angiogenesis occurs during regeneration, and pathological angiogenesis takes place during progression of fibrosis to cirrhosis and during tumorigenesis (Kaur and Anita, 2013; Gracia-Sancho et al., 2019). During both physiological and pathological angiogenesis, cellular cross-talk among several liver cell types such as sinusoidal endothelial cells (LSECs), hepatic stellate cells (HSCs), and hepatocytes orchestrates the angiogenic response in liver. Along with LSECs, bone marrow (BM)-derived endothelial progenitor cells (EPCs) are now well-reported to contribute toward post-natal vasculogenesis/angiogenesis (Asahara et al., 1997; Shi et al., 1998; Marrone et al., 2016). In response to tissue ischemia or traumatic injury, BM-derived EPCs are mobilized into the peripheral blood, migrate to sites of injured endothelium, and henceforth participate into endothelial differentiation and repair (Takahashi et al., 1999; Gill et al., 2001; Kawamoto et al., 2001; Balaji et al., 2013).

Endogenously, these cells express mixed markers present on hematopoietic stem cells and mature endothelial cells such as Vegfr2 and CD34, but do not express CD45 (Kaur and Bajwa, 2014). In culture, they can be grown as early EPCs (<14 days) or late EPCs (>14 days). Importantly, cultured EPCs express endothelial markers including vWF and eNOS (Hirschi et al., 2008). Previous studies have demonstrated that an intraperitoneal administration of EPCs in animal models of dimethylnitrosamine (DMN)- and carbon tetrachloride (CCl_4_)-induced liver injuries promotes liver regeneration and inhibits progression of liver fibrosis (Fadini et al., 2012; Nakamura et al., 2012). In comparison with the untreated animals, animals receiving EPC therapy are shown to have enhanced expression of regeneration markers, hepatocyte growth factor (HGF), tumor growth factor alpha (TGF-α), epidermal growth factor (EGF), and vascular endothelial growth factor (VEGF) and a decreased expression of fibrotic markers, alpha smooth muscle actin (α-SMA), caveolin, and endothelin-1 (Nakamura et al., 2012). The EPC-treated animals also exhibit improvements in liver function parameters including transaminases, total bilirubin, total protein, and albumin (Nakamura et al., 2007, 2012). EPC treatment in CCl4 rats has also been associated with a reduction in portal venous pressure, an increase in portal blood flow, and also an upregulated expression of endothelial nitric oxide synthase (eNOS) and VEGF (Nakamura et al., 2007).

In our previous study, we have demonstrated that in comparison with healthy human subjects, the percentage and proliferation of circulating EPCs are markedly increased in patients with cirrhosis. In these patients, cirrhotic EPCs interact, stimulate the LSECs, and enhance *in vitro* angiogenesis (Sakamoto et al., 2013). In another study, we have reported that BM-EPCs transverse to the liver during CCl4-induced liver injury. We have also shown through *in vitro* studies that EPCs activate HSCs and possibly contribute to *in vivo* fibrosis (Kaur et al., 2012). In this study, we sought to investigate the effect of cirrhotic EPCs on the phenotype and functions of LSECs and HSCs *in vivo* in bile duct models (BDL) of liver fibrosis, that most closely resemble end-stage human liver cirrhosis in many aspects.

## Materials and Methods

### Development of Experimental Animal Models of Cirrhosis by Ligation of Common Bile Duct (BDL)

The study was carried out in male Sprague-Dawley rats. All procedures were approved by the Institutional Animal Ethics Committee (IAEC) of the Institute of Liver and Biliary Sciences New Delhi, India, and experiments were conducted in accordance with Committee for the Purpose of Control and Supervision on Experiments on Animals (CPCSEA), New Delhi, India, after approval of IAEC.

Seven-week-old male Sprague-Dawley rats weighing about 200–250 g were taken for the study. Rats were housed at a controlled temperature of 24°C under a 12-h light–dark cycle and were fed standard laboratory chow and water. The surgical procedure for BDL was done under sterile conditions as described elsewhere (Garg et al., 2017). Briefly, animals were anaesthetized with ketamine hydrochloride (100 mg/kg; Neon Laboratories Limited, India) plus midazolam (5 mg/kg; Neon Laboratories Limited, India) intraperitoneally. A mid-line incision was made, and the common bile duct was isolated. On the proximal and distal side of the common bile duct, two ligatures (using silk thread 5-0) were made. The first ligature was made below the junction of hepatic duct and the second above the entry of the pancreatic duct, and a cut was made in between the two ligatures with a fine scissor. All the animals were put for the postoperative care according to the institutional animal facility standard operating procedure. Two weeks after bile duct ligation, the rats were divided into three groups: saline-treated BDL, control EPC-treated BDL, and cirrhotic EPC-treated BDL (*N* = 8 each).

### EPC Culture and Characterization

Circulating EPCs in the peripheral blood were quantified in healthy human subjects and cirrhotic patients (*N* = 8 each) by fluorescent-activated cell sorting (FACS). The characteristics of the cirrhotic patients are given in Supplementary Table S1. A total of 2–3 ml of whole blood was used for the isolation of peripheral blood mononuclear cells (PBMCs) by Ficoll method using density centrifugation (Histopaque 1077; Sigma-Aldrich, United States). After RBC lysis, using 1× RBC lysis buffer (150 mM NH_4_Cl, 10 mM KHCO_3_, 0.1 mM EDTA) for 10 min at room temperature, an equal amount of 1× PBS was added. The samples were then centrifuged at 300 × *g* at room temperature. The resulting cell pellet was washed and re-suspended in the appropriate FACS buffer (PBS, 2 mM EDTA, 2% FBS) for further cell surface staining. About 3–4 × 10^6^ cells were stained with the antibodies, anti-human FITC-CD34 (1:100), and anti-human APC-Vegfr2/Flk-1 (1:100) in PBS for 30 min at 4°C (Supplementary Table S2) (Kaur and Bajwa, 2014). The cells were then fixed with 4% PFA in PBS and analyzed by BD FACS Aria III (BD Biosciences and DIVA software). A minimum of 100,000 events were acquired for each sample. To nullify non-specific binding, CD34 and Vegfr2 antibodies (Santa Cruz Biotechnology) without any flourophores were used as negative controls).

For culture assays, circulating EPCs were further isolated and expanded *ex vivo* from patients with cirrhosis irrespective of the etiology (*N* = 10) and healthy controls (*N* = 10) as previously described (Sakamoto et al., 2013). Briefly, PBMCs were isolated from a 12–15 ml blood sample by density centrifugation (Histopaque 1077, Sigma-Aldrich, United States). After washing with PBS and RBC lysis, PBMCs (1 × 10^6^ cells/cm^2^) were seeded on fibronectin-coated 6-well plates (Nunc) in IMDM (Sigma-Aldrich) supplemented with 20% FBS (Sigma-Aldrich). For expansion, the non-adherent cells were collected after 48 h, washed and replated onto a fibronectin-coated 6-well plate with the complete medium containing IMDM supplemented with 10% FBS, 10 ng/ml VEGF, 4 ng/ml fibroblast growth factor-2 (FGF-2) and 10 ng/ml EGF (US Biologicals, United States) and antibiotics (100 U/ml penicillin and 100 μg/ml streptomycin). Medium change was done every 3 days. Adherent EPC-colonies were stained for the uptake of DiI-labeled acetylated low-density lipoprotein (acLDL, Invitrogen, United States) and binding of FITC-conjugated Ulex europaeus agglutinin I (UEA-1, Sigma) after 7 days of cell culture as earlier described (Garg et al., 2017). The stained cells were visualized under an inverted Nikon fluorescent microscope. The identity of human EPCs at day 8 of culture was also confirmed by immunofluorescence using rabbit anti-human VEGFR2 and CD34 antibodies (1: 200, Supplementary Table S2, Santa Cruz Biotechnology, Santa Cruz, CA, United States).

The adherent cells were washed with PBS and fixed in 4% paraformaldehyde for 10 min. After washing, non-specific binding was blocked with 2% BSA in PBS for 10 min. Primary antibody diluted in PBS containing 1% BSA was then added and incubated for 60 min. The cells were washed thrice with PBS and incubated for 30 min with diluted secondary antibody conjugated with a rhodamine-conjugated anti-rabbit antibody (1:500). After washing, the cells were counterstained with Ho33342 dye for 5–10 min. The cells were further washed in PBS for 5 min, mounted on a glass slide and examined under a Nikon fluorescence microscope.

### Cell Labeling

To detect the transplanted EPCs in cirrhotic animal livers, cells were pre-labeled with a green fluorescent marker, carboxyfluorescein succinimidyl ester (CFSE, Sigma Aldrich). Briefly, after thorough washing with DMEM (without FBS), 1 × 10^6^ cells/500 μl cells were labeled with 10 μM CFSE and incubated at 37°C for 15 min in a water bath.

### Transplantation of EPCs

**FIGURE 1:**
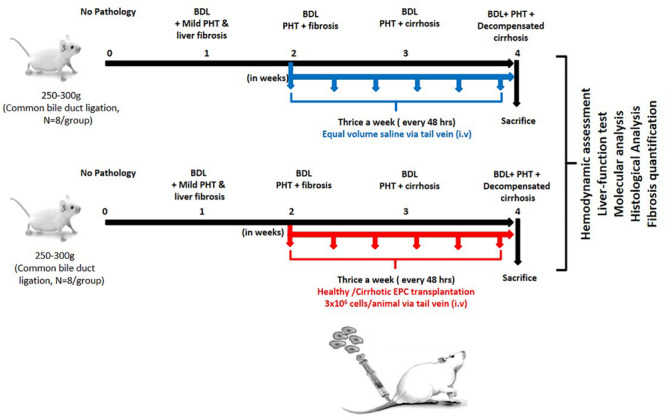
Work plan for *in vitro* and *in vivo* studies. In the *in vitro* studies, human EPCs were isolated and cultured from healthy controls (*N* = 10) and cirrhotic patients (*N* = 10). In the *in vivo* studies, the cultured EPCs from healthy controls and patients were transplanted into bile duct-ligated rats (*N* = 8 each) via the tail veil. Saline-treated rats served as the control group (*N* = 8).

After 7 days of culture in proper growth conditions as described above, adherent EPCs from cirrhotic patients and healthy controls (2 × 10^6^ cells) were trypsinized and suspended in 500 μl of PBS and transplanted in BDL rats (*N* = 8 each group) after 2 weeks of ligation intravenously through tail vein thrice a week for 2 weeks. Only saline was transplanted in the control group (*N* = 8) (Figure 1).

In a separate set of experiments, transplanted EPCs were traced in the hepatic tissues; EPCs from healthy controls were labeled with CFSE and then transplanted into cirrhotic rats. These rats were sacrificed after 1 week of EPC transplantation, and CFSE labeling was analyzed in liver tissues for the detection of EPCs.

### Evaluation of Hepatic Fibrosis

Livers from rats of all the groups were collected after cell transplantation, fixed in 10% formalin, embedded in paraffin wax, and thin sections measuring 2.5–3 μm in thickness were prepared. Sections were stained with hematoxylin–eosin and Masson’s trichrome for quantification of hepatic fibrosis. Pictures were taken and analyzed using a microscope equipped with a digital camera. Eight fields were randomly selected, and fibrosis grading was assigned by a third person blindly in all the groups.

### Gene Expression Analysis by Real-Time PCR

Total RNA from the liver tissues was isolated by using Nucleopore kit (Genetix Biotech Asia Pvt Ltd., India) as per manufacturer’s instructions. RNA was quantified at 260/280 nm with Thermo Scientific Nanodrop 2000 Spectrophotometer. First strand cDNA was synthesized from 1 μg of total RNA with reverse transcriptase (Thermo Scientific Verso cDNA synthesis kit) according to the manufacturer’s instructions. Quantitative real-time PCR was carried with SYBR green PCR master mix (Fermentas Life Sciences) on the ViiA7 PCR system (Applied Biosystems, United States). The following cycling parameters were used: start at 95°C for 5 min, denaturing at 95°C for 30 s, annealing at 60°C for 30 s, elongation at 72°C for 30 s, and a final 5 min extra extension at the end of the reaction to ensure that all amplicons were completely extended and repeated for 40 amplification cycles. Relative quantification of expression of relevant genes was done using the ΔΔCt method after normalization to the expression of the housekeeping gene, GAPDH. The genes and primer pairs are given in Table 1.

**TABLE 1:** List of primers.

**Gene**	**Forward Primer**	**Reverse Primer**
VEGFA	ACCTCCACCATGCCAAGT	TAGTTCCCGAAACCCTGA
bFGF	CCAGTTGGTATGTGGCACTG	CAGGGAAGGGTTTGACAAGA
VEGFR2	GTGATTGCCATGTTCTTCTGGC	TCAGACATGAGAGCTCGATGCT
CXCR4	TCCTGCCCACCATCTATTTTATC	ATGATATGCACAGCCTTACAT
GAPDH	CTGCACCACCAACTGCTTAC	CAGAGGTGCCATCCAGAGTT

### *In vivo* Hemodynamic Analysis

All rats had free access to food and water until 12 h before the study. Methods for the hemodynamic evaluation in portal hypertensive rat models have been extensively described in previous studies (Kountouras et al., 1984). Briefly, animals were anesthetized and the body temperature was maintained at 37 ± 0.5°C. Portal pressure (PP; mmHg; ileocolic vein), mean arterial pressure (MAP, mmHg; femoral artery), portal blood flow (PBF; mL/min; portal vein as close as possible to the liver), and superior mesenteric artery blood flow (SMABF; mL/min; superior mesenteric artery) were estimated by perivascular ultrasonic transit-time flow probes connected to a flow meter (Transonic Systems, Ithaca, NY, United States) and recorded by a PowerLab data acquisition and analysis apparatus (8/35). Data were analyzed by the Chart v5.01 software (AD Instruments). Hepatic vascular resistance (HVR, mmHg/mL⋅min⋅g^–1^) was calculated. At the end of the hemodynamic study, serum samples from all the rats were collected from inferior vena cava (IVC) for subsequent biochemical analysis.

### Serum Biochemical Analysis of Liver Function

At the time of sacrifice, serum samples from EPC transplanted BDL rats as well as from saline-treated BDL rats were collected from IVC to further evaluate alanine aminotransferase (ALT), aspartate aminotransferase (AST), bilirubin and microalbumin levels by the standard hospital protocols.

### Immunohistochemical Staining

The liver tissue sections were fixed in 10% buffered formalin solution for 24 h, embedded in paraffin wax, and thin sections measuring 2.5–3 μm in thickness were prepared. They were deparaffinized with xylene following gradual hydration with alcohol series. They were thoroughly rinsed with running tap water. Antigen retrieval was performed with citrate buffer/Tris EDTA (pH 6 and 9). Blocking of endogenous peroxidase was done in 3% hydrogen peroxide (H_2_O_2_)/H_2_O_2_ containing buffered solution of casein and sodium azide (pH 7.6) for 10 min at room temperature to avoid non-specific binding of secondary antibodies. Intrinsic peroxidase was inactivated for 10 min with 3% H_2_O_2_ and rinsed with Tris buffered substrate (TBS, 1/15 mol/l, pH 7.6).

The sections were incubated overnight at 4°C with α-SMA (BioGenex, United States, pH 6, Ready to use), CD-31 (BioGenex, United States, pH 6, Ready to use), TGF-β (Santa Cruz Biotechnology, United States, 1:200) as primary antibodies (Supplementary Table S2) followed by a reaction for 30 min at 20°C using a biotinylated secondary antibody (Super Sensitive polymer HRP IHC Detection System, BioGenex, Fremont, United States). After washing, the tertiary antibody (Super Sensitive polymer HRP IHC Detection System, BioGenex, Fremont, United States) was used for 20 min. Sections were rinsed first with TBS and then under running tap water. Then, mixed solution of 3, 3-diaminobenzidine tetra hydrochloride (DAB) substrate (in dark) was used for color development (visualization) of the reaction product. Sections were further counterstained with hematoxylin for 1 min, dehydrated and mounted with DPX and observed under the microscope.

### Protein Expression Analysis by Western Blotting

Samples of shock-frozen livers were homogenized in a buffer containing 25 mM Tris/HCl, 5 mM ethylenediamine tetraacetic acid, 10 μM phenylmethanesulfonyl fluoride, 1 mM benzamidine, and 10 μg/mL leupeptin. Samples were diluted with sample buffer. Determination of proteins in the homogenates was performed with Bradford (Sigma) and pre-diluted protein assay standards (BSA set kit, thermo scientific, United States). Samples (50 μg of protein/lane) were subjected to sodium dodecyl sulfate polyacrylamide gel electrophoresis (SDS-PAGE; 15% gels) for α-SMA and CD31, and proteins were blotted on PVDF membranes charged with methanol. To ensure equal protein loading, Ponceau-S staining was performed. The membranes were blocked with 5% BSA, incubated with primary antibodies α-SMA (1:1000) and CD31 (1:200) and thereafter with corresponding secondary peroxidase-coupled antibodies (Supplementary Table S2). Blots were developed with enhanced chemiluminescence Pierce, ECL plus western blotting substrate (Thermo scientific, United States). Intensities of the resulting bands on each blot were compared densitometrically with image J software.

### Statistical Analysis

Using SPSS software, statistical analysis was performed. Results are expressed as mean + standard deviation. Comparisons between groups were performed with the Student’s *t*-test for unpaired data. Differences were considered significant at a *P*-value < 0.05.

## Results

### Characterization of Human EPCs *in vitro* and *in vivo*

**FIGURE 2:**
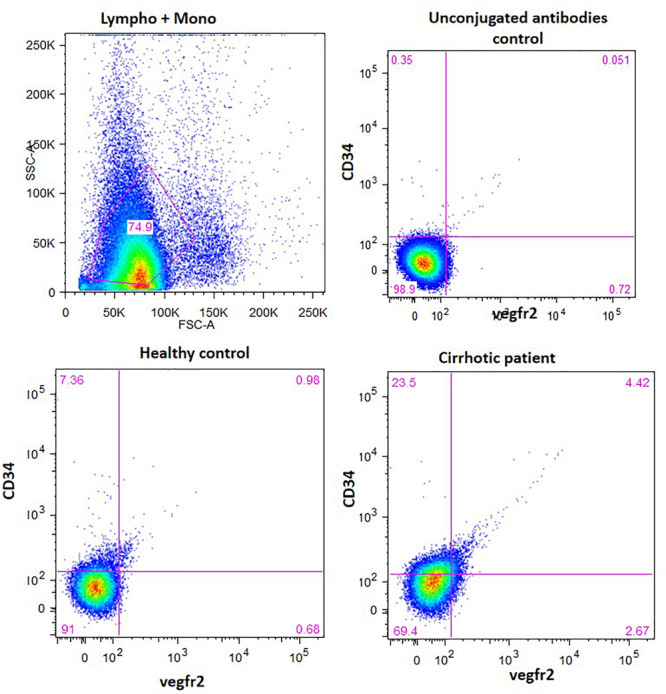
EPC enumeration in whole blood by measuring the percentage of CD34 and vergfr2 dual positive cells in the lymphocyte and monocyte gated cells. The figure shows dot plots of dual stained cells in healthy controls and cirrhotic patients. Unconjugated antibodies without flourophores were used as controls.

**FIGURE 3:**
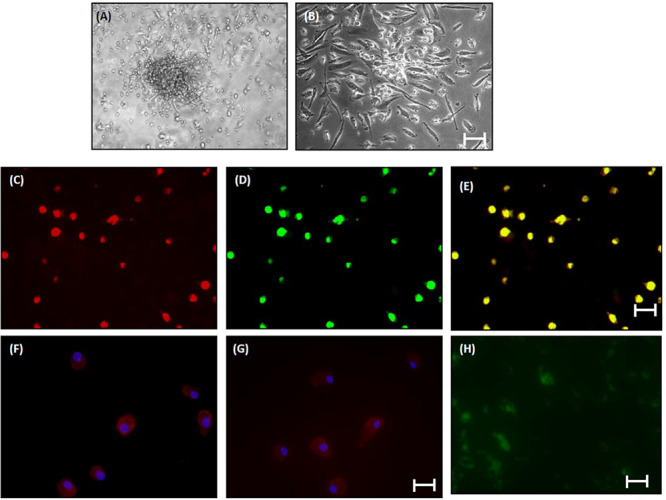
Characterization of human EPCs from healthy controls (20x) **(A)** and **(B)** phase-contrast micrograph of culture-enriched endothelial progenitor cells (EPC-CFUs) on 7th day and 9th day in controls. **(C)** DiI-acLDL uptake by EPCs. **(D)** FITC-UEA lectin binding by EPCs (explained in the section “Materials and Methods”). **(E)** Overlay of **(C)** and **(D)**. **(F)** Immunofluorescence characterization of cultured EPCs with anti-human VEGFR2. **(G)** Anti-human CD34. Cells were counterstained with Hoechst dye. **(H)** *In vivo* localization of CFSE-labeled control EPCs in liver tissue sections after 24 h of transplantation.

The percentage of CD34-vegfr2 dual positive EPCs in blood was increased in patients with cirrhosis as compared to the healthy subjects (Figure 2). At day 2–3, large round adherent cells started appearing from plated human PBMCs, which then became spindle-shaped at around day 7–9 (Figures 3A,B). The attached cells after day 7 stained positive for DiI-acetylated LDL (red fluorescence) and FITC-UEA-1 lectin (green fluorescence) (Figures 3C–E). After 7 days in culture, the adherent cells also stained positive for specific EPC surface proteins, vegfr2 (Figure 3F) and CD34 (Figure 3G). EPCs from patients with cirrhosis also behaved in a similar fashion in culture (results not shown).

*In vivo*, EPCs labeled with CFSE (green fluorescence) were observed surrounding the portal tracts, fibrous septa, and hepatic lobules in the BDL rats, 24 h after EPC transplantation (Figure 3H). We did not observe any infiltrated cells with green fluorescence in the saline-infused BDL rat livers (data not shown).

### Collagen Deposition in EPC-Transplanted BDL Rats

**FIGURE 4:**
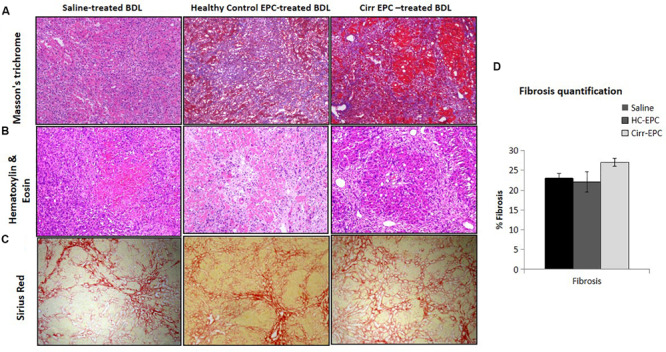
Assessment of liver fibrosis (10x) in saline-treated BDL, healthy control (HC), and cirrhotic (cirr) EPC-transplanted BDL rats by **(A)** Masson’s trichome staining, **(B)** H and E staining, **(C)** Sirus red staining, and **(D)** fibrosis quantification.

In saline-treated BDL rats, hepatic necrosis and mild fibrosis with some viable inflammatory cells were observed. Peribiliary and interstitial collagen deposition was evident in all groups of BDL rats, as shown by positive Masson’s trichrome staining (Figures 4A,B). There was an increase of portal fibrosis (grade 3) to marked cirrhosis (grade 4) in cirrhotic-EPC-treated rats as compared to healthy EPC-treated and saline-treated BDL rats (Figures 4A–D). Liver collagen staining as determined by Sirius red was less in the liver in saline-treated and healthy control-EPC treated rats in comparison with cirrhotic-EPC-treated rats (Figures 4C,D).

### Intrahepatic Angiogenesis and Fibrosis in EPC-Transplanted BDL Rats

**FIGURE 5:**
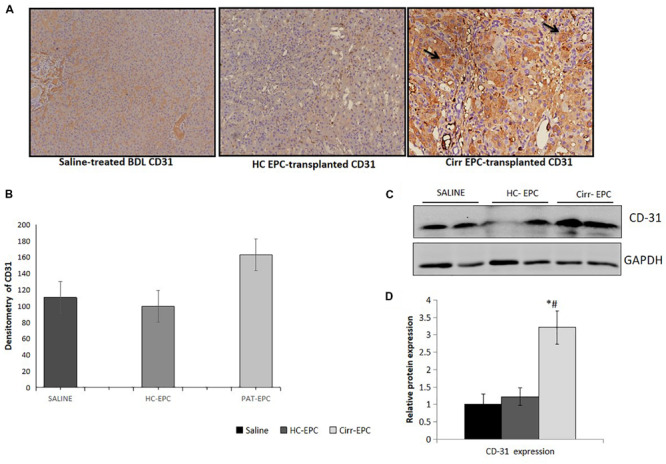
Histochemical staining of liver tissues sections (10x) in saline-treated, healthy (HC), and cirrhotic (PAT) EPC-transplanted BDL rats for angiogenic markers. **(A)** CD31, **(B)** Densitometric analysis of CD31, **(C)** Representative western blots of CD31, and **(D)** Densitometric analysis of western blot in saline-treated, control, and cirrhotic EPC-transplanted BDL rats (*N* = 8). *P < 0.05 vs. saline, #, vs. HC-EPC.

The expression of CD31 was less in healthy EPC- and saline-treated rat liver sections in both immunohistochemistry and western blot studies. There was an increase in the expression of LSEC differentiation and angiogenesis marker, CD31 in cirrhotic EPC-treated rats as compared to healthy EPC- and saline-treated rats (*P* < 0.05 for each). In cirrhotic EPC-transplanted rats, the expression of CD31 was majorly seen in portal areas/peri-portal regions and fibro-septae suggesting neovascularization in these areas (Figures 5A–D).

**FIGURE 6:**
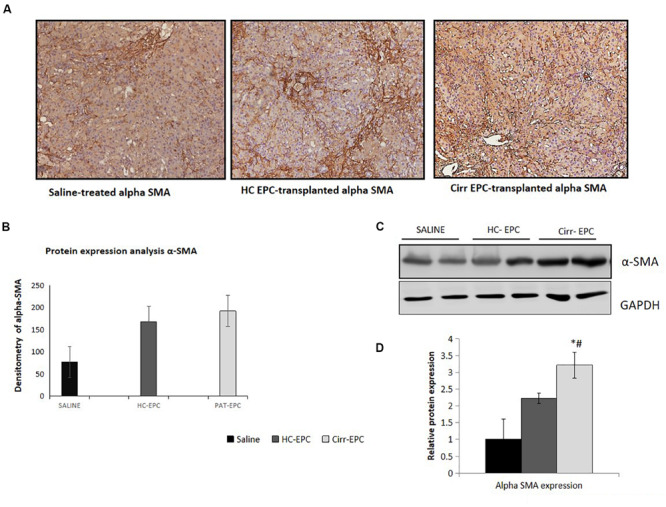
Histochemical staining of liver tissues sections (10x) in saline-treated, healthy control, and cirrhotic EPC-transplanted rats for fibrosis markers. **(A)** Alpha-SMA, **(B)** Densitometric analysis of alpha-SMA, **(C)** Representative western blots of alpha-SMA, and **(D)** Densitometric analysis of western blots of alpha-SMA in saline-treated, control, and cirrhotic EPC-transplanted rats. *P < 0.05 vs. saline, #, vs. HC-EPC.

Increased α-SMA positive cells paralleled the development of increased fibrosis in BDL rats. Histochemical and densitometric analysis of the western blots showed that the expression of the fibrosis marker, alpha-SMA present on activated HSCs was significantly higher in cirrhotic EPC-treated rats as compared to the saline- and healthy-EPC treated rats (*P* < 0.05 for each) (Figures 6A–D). The expression of TGF-β, a fibrosis marker, was also increased in the liver sections of cirrhotic EPC-treated rats and control-EPC treated rats as compared to the saline-treated rats (Supplementary Figure S1). To ascertain if patient EPCs specifically activated HSCs in culture, we set up co-cultures between HSC cell lines (LX2) and conditioned media (CM) from EPCs (both healthy and cirrhotic). Results showed that in the presence of patient EPCs, there was a maximum proliferation of LX2 cells, much higher than that observed with control EPCs or negative control (media only) (Supplementary Figures S3A,B). Also secretion of an important angiogenic factor, basic fibroblast growth factor (bFGF), was significantly higher in HSCs co-cultured in the presence of CM from cirrhotic patients’ EPCs in comparison to that with HSCs with CM from control or healthy EPCs (Supplementary Figure S3C).

### Hepatic Hemodynamics in EPC-Transplanted BDL Rats

Bile duct ligation cirrhotic animals exhibited portal hypertension when compared to control rats (Table 2). The BDL cirrhotic rats transplanted with cirrhotic EPCs exhibited statistically significant higher portal pressure than rats transplanted healthy EPCs or receiving vehicle (17.2 ± 2.1 vs. 13.8 ± 2.2 mmHg; +25%; *P* = 0.003). We did not observe any change in PP in healthy EPC transplanted rats in comparison with vehicle treated rats (14.5 ± 2.1 vs. 13.8 ± 2.2 mmHg; +5%; *P* = 0.91). Also, we did not observe any change in PBF. The increment in PP was not associated with change in PBF, thus suggesting that portal hypertension aggravation in cirrhotic EPC transplanted rats derived from an increment in the HVR (cirrhotic EPCs rats: +25%) in comparison with healthy EPC transplanted rats (*P* < 0.05). MAP, SMABF, and HR were not modified by cell transplantation (Table 3).

**TABLE 2:** Hemodynamic parameters.

**Characteristics hemodynamic parameters**	**Experimental groups**		
	**Healthy control**	**BDL Veh**	***P*-value**
*N*	6	8	–
MAP (mmHg)	11310	749	*P* < 0.05
PP (mmHg)	7.50.9	14.52.1	*P* < 0.05
PBF (ml/min)	14.23.6	18.98.6	*P* < 0.05
HVR (mmHg/ml⋅min⋅g^–1^)	2.50.6	22.514.3	*P* < 0.05
HR (beats/min)	35449	34533	NS
Liver weight	113.2	256.6	*P* < 0.05
Body weight	34065	41095	NS

**TABLE 3:** Hemodynamic parameters.

**Characteristics hemodynamic parameters**	**Experimental groups**			
	**BDL Veh**	**BDL + Healthy EPC**	**BDL + Cirrhotic EPC**	***P*-value**
*N*	8	8	8	–
MAP (mmHg)	749	689.7	7128	NS
PP (mmHg)	14.52.1	13.82.2	17.22.1	*^#^*P* < 0.05
PBF (ml/min)	18.98.6	17.14.8	20.67.2	NS
HVR (mmHg/ml⋅min⋅g^–1^)	22.514.3	20.17.2	25.613	*^#^*P* < 0.05
HR (beats/min)	34533	333147	42637	NS
Liver weight	256.6	27.68.4	27.44.9	NS
Body weight	41095	398134	40175	NS

*P < 0.05 Cirrhotic EPC vs. Veh; ^#^*P* < 0.05 Cirrhotic EPC vs. Healthy EPC.

### Liver Functions in EPC-Treated BDL Rats

Serum from the untreated and EPC-treated rats was collected for the analysis of liver functions including the estimation of the levels of urea, total bilirubin, glucose, micro albumin, and alanine transaminase. We did not observe any significant difference in the levels of urea, bilirubin, glucose, albumin, ALT, and AST in EPC-treated BDL rats as compared to saline-treated BDL rats (Supplementary Figure S2).

### Angiogenic Gene Expression in EPC-Treated BDL Rats

**FIGURE 7:**
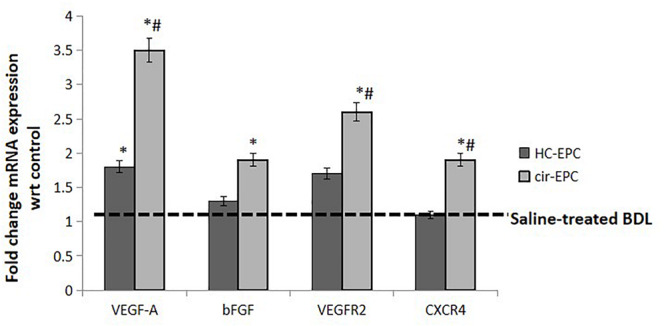
mRNA expression of genes in liver tissue samples of healthy and cirrhotic EPC-transplanted rats. Dotted line represents control showing gene expression in Huh7 cells treated with BSA. Data represent mean ± SD, (*n* = 4). *P < 0.05 vs. saline, ^#^*P* < 0.05 vs. healthy EPC-treated rats.

Next, we studied the effect of healthy and cirrhotic EPC transplantation on angiogenic gene expression in the liver tissues. With respect to saline-treated rats, the expression of VEGFA gene was markedly upregulated in the livers of both healthy and cirrhotic EPC transplanted rats. However, the expression of other genes including VEGFR2, bFGF, and CXCR4 was enhanced only in cirrhotic EPC-transplanted rats in comparison with both saline- and healthy EPC-treated rats (Figure 7).

## Discussion

**FIGURE 8:**
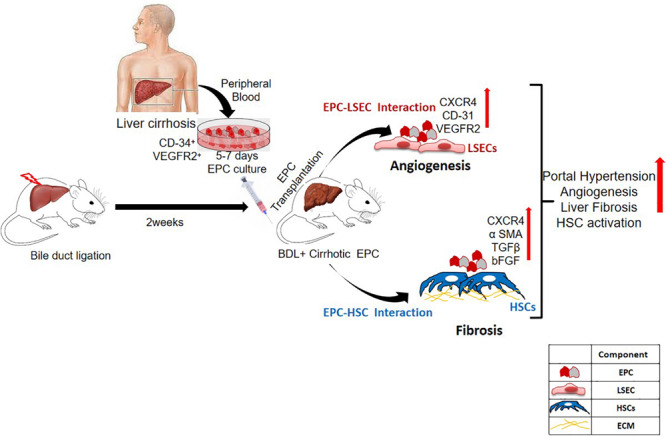
Overall summary of the study. Transplantation of cirrhotic EPCs (CD34+-vegfr2+) in BDL rats interacts with LSECs and HSCs *in vivo* and increases hepatic angiogenesis, and fibrosis further aggravates portal hypertension.

Endothelial progenitor cells have been implicated in both injury and repair. Previous studies have highlighted the regenerative role of healthy EPCs during liver injury. These studies have shown their role in restoring vascular density and promoting hepatic regeneration in the injured livers (Fadini et al., 2012; Nakamura et al., 2012). However, as angiogenesis plays a pathogenic role in fibrosis, we hypothesized that EPCs would be pro-fibrotic as well (Medina et al., 2004, 2005; Ding et al., 2014; Tripathi et al., 2018). Our previous studies have demonstrated that during liver injury, endogenous bone marrow EPCs migrate to the liver and show strong correlation with liver fibrosis (Kaur et al., 2012). In this study, we evaluated the *in vivo* effects of healthy and cirrhotic EPCs in liver injury. We demonstrate that transplantation of cirrhotic EPCs (CD34+ vegfr2+) in BDL rats aggravates hepatic angiogenesis, fibrosis, and portal hypertension possibly via their interaction with LSECs and HSCs (Figure 8).

Our results showed an increase in CD31-postive blood vessels and angiogenesis in BDL models in comparison with the control rats as has been reported earlier (Fadini et al., 2012; Nakamura et al., 2012). There was a further increase in the number of CD31-positive blood vessels in cirrhotic EPC-treated BDL rats as compared to healthy EPC- and saline-treated BDL rats. Healthy liver vessels or LSECs are fenestrated and normally do not express CD31. An increased expression of CD31 is indicative of LSEC differentiation and plays a pivotal role in the activation of HSCs (Lee and Friedman, 2011). We also observed a significant enhancement in the hepatic expression of angiogenic growth factors and receptors such as VEGF, bFGF, and receptors including VEGFR2 and CXCR4 in cirrhotic EPC-treated BDL rats as compared to healthy EPC-treated rats. This suggests that the proangiogenic activity of cirrhotic EPCs is excessive and may induce the formation of abnormal vessels in the already cirrhotic liver via upregulation of signals such as VEGF and bFGF. Our results corroborate the findings of earlier studies that bFGF, VEGF, and VEGFR2 play key roles in the angiogenesis of the cirrhotic liver (Rosmorduc et al., 1999; Medina et al., 2005; Lee and Friedman, 2011; Xie et al., 2012). Our previous study also demonstrated that cirrhotic EPCs secrete higher levels of bFGF and VEGF in culture and exhibit greater angiogenic impact on LSECs in culture. It has also been shown earlier that a predominance of CXCR4 expression of LSECs shifts the pro-regenerative response toward the pro-fibrogenic response during liver injury (Medina et al., 2004). Activated LSECs are known to modulate HSCs that then mutually promote the function of each other, leading to liver fibrosis (Lee and Friedman, 2011). We observed a significant increase in the fibrogenic markers, α-SMA and TGF-β in cirrhotic-EPC treated BDL rats, as compared to the saline- and healthy EPC-treated rats, suggesting that the formation of abnormal vessels by cirrhotic EPCs facilitates liver fibrogenesis or even vice versa. CM from cirrhotic EPCs also significantly enhanced the proliferation of LX2 cells and secretion of angiogenic factor in culture more than the healthy EPCs, suggesting their direct role in HSC activation. This observation validates our previous study, where we reported that CM from EPCs increased the expression of alpha-SMA in the cultured mouse HSCs (Kaur et al., 2012).

An increase in liver angiogenesis and fibrosis causes deregulation of the hepatic hemodynamics and correlates positively with portal hypertension (Poisson et al., 2017; McConnell and Iwakiri, 2018). Our results demonstrated that pro-angiogenic cirrhotic EPCs markedly affect the vascular physiology of the liver (Gracia-Sancho et al., 2019). They enhanced the portal pressure favoring hypercontractility state, leading to increased intrahepatic vascular resistance and decreased liver perfusion. A mild increase (non-significant data) in portal pressure and a decrease in hepatic vascular resistance were observed in healthy EPC transplanted cirrhotic models as has been reported earlier (Nakamura et al., 2007), again suggesting that healthy and cirrhotic EPCs have significant differences in their properties and that cirrhotic EPCs have a detrimental impact on portal pressures and blood flow in the liver in comparison with the healthy EPCs.

We observed no changes in the liver functions including transaminases, total bilirubin, total protein, and albumin either in healthy or cirrhotic EPC-transplanted rats. It has been, however, reported in an earlier study that normal liver function parameters are restored in EPC-transplanted CCl_4_-treated rats (Garg et al., 2017). This may be due to the fact that we used BDL models in our study which is a combined model of hepatocyte and cholestatic liver injury. Unlike the CCl4-induced liver cirrhotic models, in the BDL model, extrahepatic cholestasis due to prolonged obstruction of bile flow results in even more extensive morphological and biochemical changes (Fadini et al., 2012). The low efficacy of healthy EPC treatment in our study may be attributed to an increased degree of fibrosis and deposition of extracellular matrix proteins in the BDL rats in comparison with the CCl4-treated rats. A limitation of our study is that we have transplanted human EPCs into rats. Although the adaptive immune functions of BDL rats are highly immunocompromised, these rats elicit strong innate immune responses such as activation of neutrophils, macrophages, and natural killer cells which are currently being recognized as important components in xenograft rejection (Chaignaud et al., 1994). Another limitation of the study is the small sample number of animals used to perform adequate statistical comparisons. Also, we have used a very heterogeneous group of CD34-vegfr2-positive EPCs for transplantation, and hence, it is difficult to interpret the true relevance of angiogenic EPCs (which may be a small subset) to liver fibrosis and/or improvement in functions (Fadini et al., 2012).

Overall, this study concludes that cirrhotic EPCs have enhanced angiogenic and profibrogenic functions *in vivo* as compared to the healthy control EPCs. Our findings imply that the endogenous mobilization of cirrhotic EPCs in cirrhotic patients may lead to fibrosis due to enhanced proangiogenic activity of EPCs. Hence, these patients are likely to benefit from therapies which are aimed at lowering the numbers of proangiogenic EPCs.

## Data Availability Statement

All datasets presented in this study are included in the article/Supplementary Material.

## Ethics Statement

The animal study was reviewed and approved by the Institutional Animal Ethics Committee, ILBS.

## Author Contributions

DT, MH, and HS performed all animal experiments, western blotting, and analyzed the data. DT did all the hemodynamic experiments and analysis. DT and MH wrote the manuscript. IK and CB performed immunohistochemistry of the liver tissues. PR performed RT-PCRs and data analysis. SK designed the study, performed the data analysis along with DT, and finalized the manuscript. SS gave inputs in designing of the study and editing of the manuscript.

## Conflict of Interest

The authors declare that the research was conducted in the absence of any commercial or financial relationships that could be construed as a potential conflict of interest.

## Supplementary Material

FIGURE S1 Immunohistochemical staining of liver tissues sections in saline-treated, control, and cirrhotic EPC-transplanted rats for TGF-β.

FIGURE S2 Histograms showing the effect of saline, control, and cirrhotic EPCs treatment on liver functions in BDL rats.

FIGURE S3 **(A)** Representative images (10×) of LX2 cells (HSCs) incubated with conditioned media (CM) from healthy (control) and cirrhotic patient EPCs. **(B)** Bar diagram showing absorbance of MTT assay depicting cell proliferation in different conditions. **(C)** Absorbance showing FGF levels in cells alone and co-cultured cells.

TABLE S1 Characteristics of patients used in the study.

TABLE S2 List of Antibodies used in the study.
