# Supplementary figures and images for: Cirrhotic Endothelial Progenitor Cells Enhance Liver Angiogenesis and Fibrosis and Aggravate Portal Hypertension in Bile Duct-Ligated Cirrhotic Rats
Source: Front Physiol. 2020 Jun 11;11:617. doi: 10.3389/fphys.2020.00617 (PMC7300214; doi:10.3389/fphys.2020.00617)

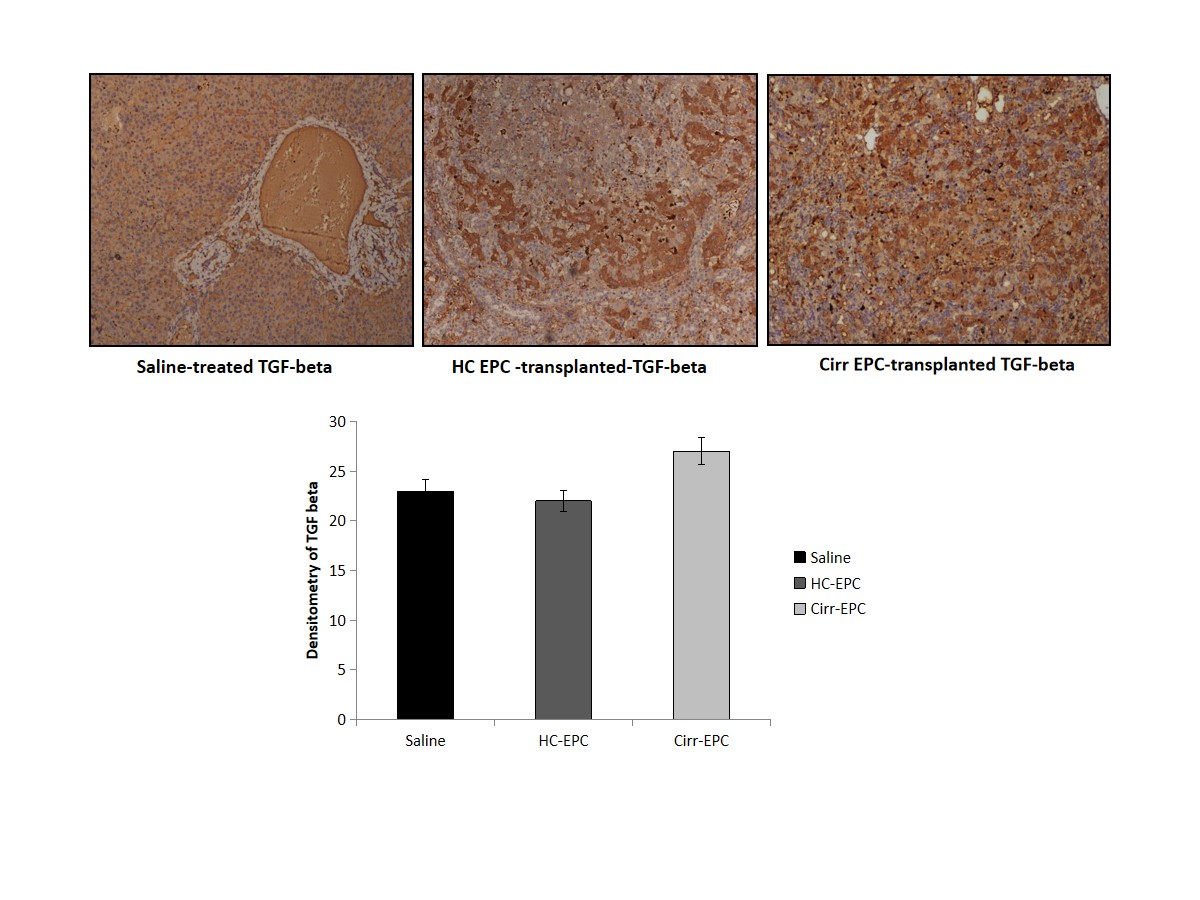

Supplement: FIGURE S1 — Immunohistochemical staining of liver tissues sections in saline-treated, control, and cirrhotic EPC-transplanted rats for TGF-β. [file Image_1.JPEG]

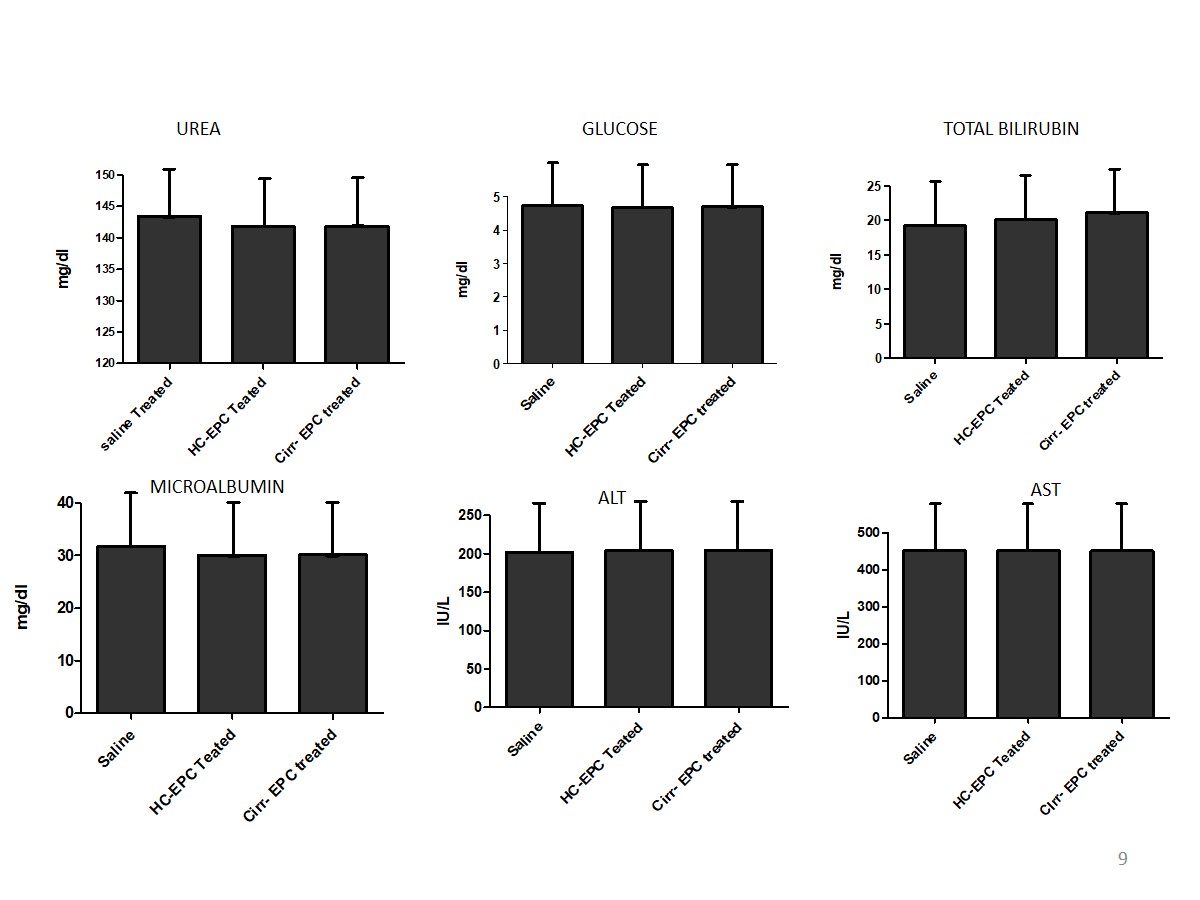

Supplement: FIGURE S2 — Histograms showing the effect of saline, control, and cirrhotic EPCs treatment on liver functions in BDL rats. [file Image_2.JPEG]

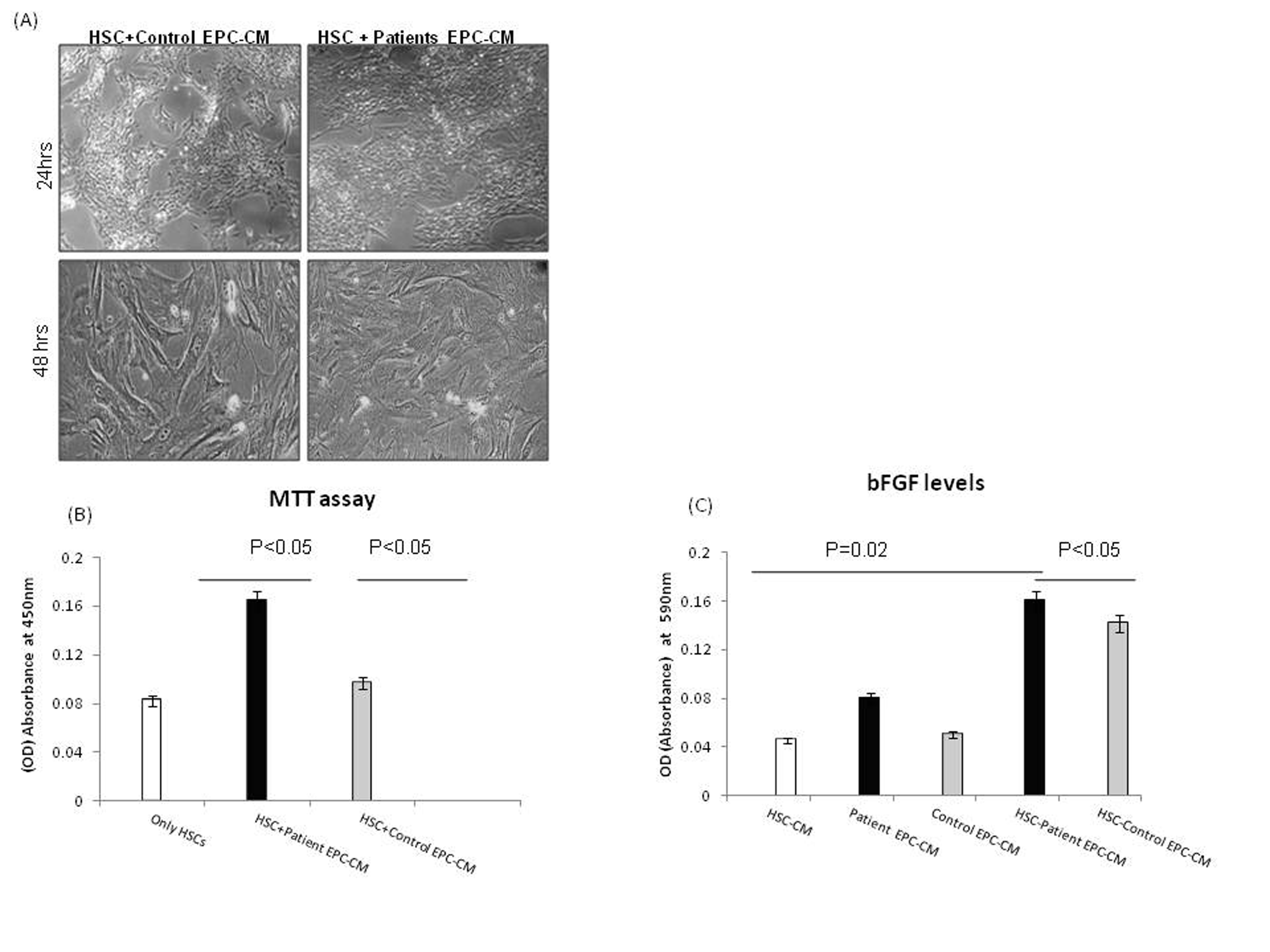

Supplement: FIGURE S3 — (A) Representative images (10×) of LX2 cells (HSCs) incubated with conditioned media (CM) from healthy (control) and cirrhotic patient EPCs. (B) Bar diagram showing absorbance of MTT assay depicting cell proliferation in different conditions. (C) Absorbance showing FGF levels in cells alone and co-cultured cells. [file Image_3.JPEG]
