# Supplementary material for: Cirrhotic Endothelial Progenitor Cells Enhance Liver Angiogenesis and Fibrosis and Aggravate Portal Hypertension in Bile Duct-Ligated Cirrhotic Rats
Source: Front Physiol. 2020 Jun 11;11:617. doi: 10.3389/fphys.2020.00617 (PMC7300214; doi:10.3389/fphys.2020.00617)
Supplement: TABLE S1 — Characteristics of patients used in the study. [file Table_1.doc]

**Supplementary Methods**

**Culture of LX2 cells and co-culture with healthy and cirrhotic EPCs:** LX-2 cells (Hepatic stellate cells) were cultured in Dulbecco’s Modified Eagle’s medium (DMEM) (Gibco) with 2% FBS (Hyclone) and 100µg/ml streptomycin and 100 IU/ml penicillin at 37℃ with 5% CO_2_. EPCs from healthy controls and patients at day 7, were washed and replenished with serum deprived (serum free) medium for 48 hours for the collection of conditioned media (CM). The CM from both cirrhotic patient EPCs and healthy control EPCs was put over LX2 cells (2×10^3^ cells/well) in 96-well culture plates and co-cultured for 24 h*.* The proliferation of HSCs was studied in presence of control and cirrhotic EPC-CM using MTT assay. For MTT assay, cells were washed after 24 h incubation, missed with MTT at a final concentration of 0.5 mg/mL and incubated for 3 hours at 37°C. MTT was removed and solubilizing solution was added. Absorbance was taken at 570nm after incubation at room temperature or 37°C for 30 minutes to 2 hours, until cells had lysed and purple crystals had dissolved. For estimating bFGF levels, after 24 hours of incubation, the levels of important pro-angiogenic factor, basic fibroblast growth factor (bFGF) was measured in the EPC-HSC co-cultures conditioned media and that of cell cultures alone by sandwich enzyme-linked immunosorbent assay (ELISA) using a commercially available kit used in accordance with manufacturer’s instructions (R&D systems Inc, USA).

**Supplementary Tables**

**Table S1:** **Characteristics of patients used in the study**

| **Parameters** | **Cirrhosis (4)** |
| --- | --- |
| **Age (mean**+SD, yr.) | 54.8 + 9.25 |
| **Gender** | 3M, 1F |
| **INR** | 1.5 + 0.5 |
| **Serum albumin (g/dL)** | 2.9 + 0.2 |
| **Total bilirubin (mg ⁄ dL)** | 2.73 + 1.7 |

Table S2: List of Antibodies used in the study

| **Antibody** | **Antibody source** | **Cat No** | **Application** |
| --- | --- | --- | --- |
| Anti-human CD34 Antibody-FITC | Santa Cruz | sc-7324 FITC | Flow cytometry |
| Human VEGFR2/KDR/Flk-1 APC-conjugated Antibody  Human VEGFR2/KDR/Flk-1 APC-conjugated Antibody | R & D systems | FAB357A-025 | Flow cytometry |
| rabbit anti-human VEGFR2 | Santa Cruz | sc-504 | Cell Immunoflourescence |
| Anti-human CD34 Antibody-Unconjugated | Santa Cruz | sc-7324 | Cell Immunoflourescence |
| Rhodamine-conjugated anti-rabbit antibody | Elabscience | E-AB-1053 | Cell Immunoflourescence |
| CD31 Antibody | BioGenex | AM241 | Immunohistochemistry |
| TGF-β Antibody | Santa Cruz | sc-130348 | Immunohistochemistry |
| α-SMA Antibody | BioGenex | AM128 | Immunohistochemistry |
| a-SMA Antibody | Santa Cruz | sc-53015 | Western Blotting |
| CD31 Antibody | Santa Cruz | sc-376764 | Western Blotting |
